# Supplementary material for: Mathematical modeling of plus-strand RNA virus replication to identify broad-spectrum antiviral treatment strategies
Source: bioRxiv. 2022 Jul 25:2022.07.25.501353. Preprint. [Version 1] doi: 10.1101/2022.07.25.501353 (PMC9347285; doi:10.1101/2022.07.25.501353)
Supplement: 1 [file NIHPP2022.07.25.501353V1-supplement-1.pdf]

bioRxiv preprint doi: https://doi.org/10.1101/2022.07.25.501353; this version posted July 25, 2022. The copyright holder for this preprint (which was not certified by peer review) is the author/funder. This article is a US Government work. It is not subject to copyright under 17 USC 105 and is also made available for use under a CC0 license.

S1 Supporting material: Model selection process.

S1 Supporting data

Drug A	Drug B	HCV	DENV	CVB3
TC formation (k_1)	-	0.96	1	1
Translation (k_2)	-	0.99	0.99	1
Polyprotein cleavage (k_c)	-	0.995	1	1
RC formation (k_{Pin})	-	0.99	1	-
RNA synthesis (k_{4p} and k_{4m})	-	0.89	0.865	0.995
Viral export (k_{Pout})	-	1	1	1
Virus assembly and release (k_p)	-	1	1	1
TC formation (k_1)	***RNA synthesis (k_{4p} and k_{4m})***	**0.76**	**0.85**	0.993
TC formation (k_1)	***RC formation (k_{Pin})***	0.85	0.99	1
Translation (k_2)	***RNA synthesis (k_{4p} and k_{4m})***	0.90	**0.85**	0.99
Translation (k_2)	***RC formation (k_{Pin})***	0.96	0.98	0.991
Polyprotein cleavage (k_c)	***RNA synthesis (k_{4p} and k_{4m})***	0.90	0.87	**0.98**
Polyprotein cleavage (k_c)	***RC formation (k_{Pin})***	0.997	0.999	1

S1 Table: Critical drug efficacy constants in mono and combination therapy and an in-silico drug administration in steady state (100 h pi). For simplicity, we assume that in combination therapy, both drugs have the same efficacy. The lowest critical drug efficacies to clear the virus-specific infection is highlighted in red (TC = translation complex, RC = replicase complex)

bioRxiv preprint doi: https://doi.org/10.1101/2022.07.25.501353; this version posted July 25, 2022. The copyright holder for this preprint (which was not certified by peer review) is the author/funder. This article is a US Government work. It is not subject to copyright under 17 USC 105 and is also made available for use under a CC0 license.

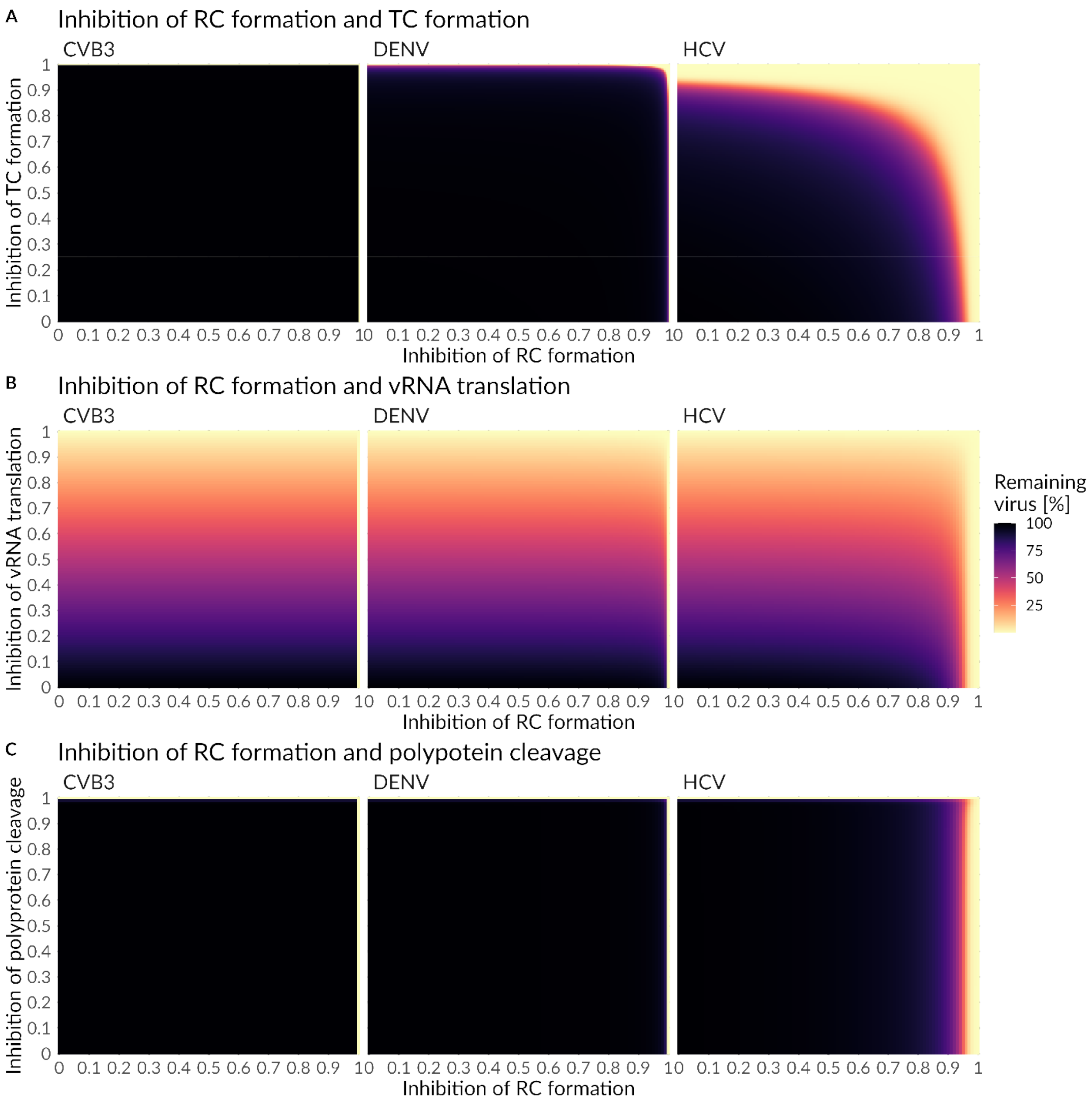

S1 Figure: Combined drug effect on ***A)*** *replicase complex (RC) formation and formation of translation complex (TC)* ***B)*** *replicase complex (RC) formation and polyprotein cleavage and* ***C)*** *replicase complex (RC) formation and vRNA translation and drug administration in steady state (100 h pi). A successful drug treatment leads to a more than 99% viral eradication (light yellow), while an ineffective drug treatment leads to 100% remaining virus (black).*

bioRxiv preprint doi: https://doi.org/10.1101/2022.07.25.501353; this version posted July 25, 2022. The copyright holder for this preprint (which was not certified by peer review) is the author/funder. This article is a US Government work. It is not subject to copyright under 17 USC 105 and is also made available for use under a CC0 license.

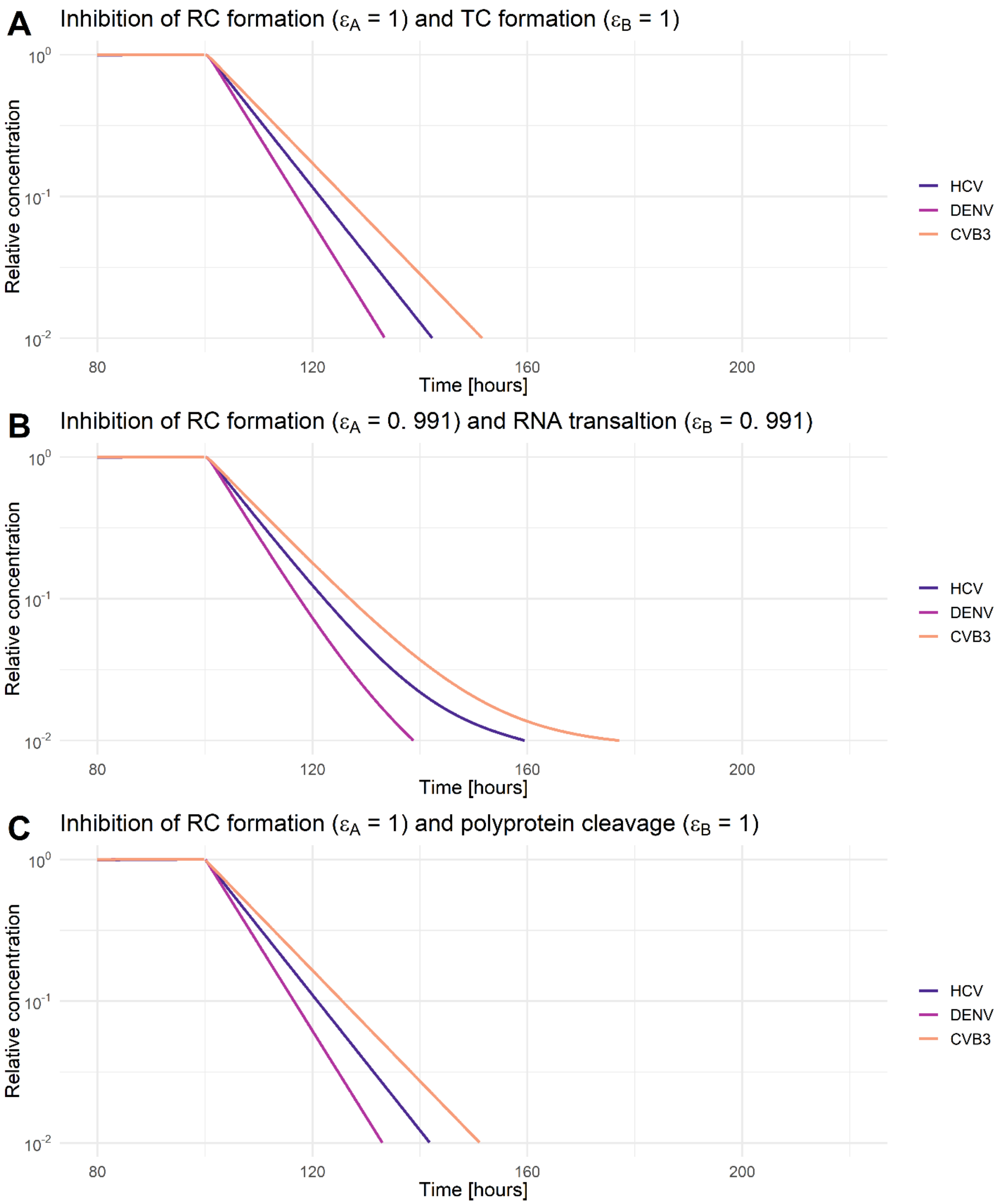

S2 Figure: Relative virus decay under combination therapy that clears HCV, DENV, and CVB3 infections. A combined drug effect on ***A)*** *formation of replicase complex (RC) and formation of translation complex (TC),* ***B)*** *formation of replicase complex (RC) and translation, and* ***C)*** *formation of replicase complex (RC) and polyprotein cleavage. Initiation of treatment was in steady state (100 h pi). The drug efficacy*

bioRxiv preprint doi: https://doi.org/10.1101/2022.07.25.501353; this version posted July 25, 2022. The copyright holder for this preprint (which was not certified by peer review) is the author/funder. This article is a US Government work. It is not subject to copyright under 17 USC 105 and is also made available for use under a CC0 license.

constant (ε_A and ε_B) were chosen as minimal efficacies to clear all three viruses. For comparability, virus-specific concentrations in steady state have been normalized to their virus-specific pre-treatment steady state concentration. A successful drug treatment leads to a more than 99% viral eradication (light yellow), while an ineffective drug treatment leads to 100% remaining virus (black) (see S1 Supporting data).
